# The Development and Validation of a Novel Nanobody-Based Competitive ELISA for the Detection of Foot and Mouth Disease 3ABC Antibodies in Cattle

**DOI:** 10.3389/fvets.2018.00250

**Published:** 2018-10-12

**Authors:** Sigal Gelkop, Ariel Sobarzo, Polina Brangel, Cécile Vincke, Ema Romão, Shlomit Fedida-Metula, Nick Strom, Irene Ataliba, Frank Norbet Mwiine, Sylvester Ochwo, Lauro Velazquez-Salinas, Rachel A. McKendry, Serge Muyldermans, Julius Julian Lutwama, Elizabeth Rieder, Victoria Yavelsky, Leslie Lobel

**Affiliations:** ^1^The Shraga Segal Department of Microbiology, Immunology and Genetics, Faculty of Health Sciences, Ben-Gurion University of the Negev, BeerSheba, Israel; ^2^London Centre for Nanotechnology and Div. of Medicine, University College London, London, United Kingdom; ^3^Laboratory of Cellular and Molecular Immunology, Vrije Universiteit Brussel, Brussels, Belgium; ^4^Virology Division, Kimron Veterinary Institute, Beit Dagan, Israel; ^5^Department of Arbovirology, Emerging and Re-emerging Infection Uganda Virus Research Institute, Entebbe, Uganda; ^6^College of Veterinary Medicine, Animal Resources and Biosecurity (COVAB), Makerere University, Kampala, Uganda; ^7^Foreign Animal Disease Research Unit, United States Department of Agriculture Plum Island Animal Disease Center, Agricultural Research Service (USDA), New York, NY, United States

**Keywords:** foot and mouth disease, non-structural proteins, nanobodies, antibodies, ELISA

## Abstract

Effective management of foot and mouth disease (FMD) requires diagnostic tests to distinguish between infected and vaccinated animals (DIVA). To address this need, several enzyme-linked immunosorbent assay (ELISA) platforms have been developed, however, these tests vary in their sensitivity and specificity and are very expensive for developing countries. Camelid-derived single-domain antibodies fragments so-called Nanobodies, have demonstrated great efficacy for the development of serological diagnostics. This study describes the development of a novel Nanobody-based FMD 3ABC competitive ELISA, for the serological detection of antibodies against FMD Non-Structural Proteins (NSP) in Uganda cattle herds. This in-house ELISA was validated using more than 600 sera from different Uganda districts, and virus serotype specificities. The evaluation of the performance of the assay demonstrated high diagnostic sensitivity and specificity of 94 % (95 % CI: 88.9–97.2), and 97.67 % (95 % CI: 94.15–99.36) respectively, as well as the capability to detect NSP-specific antibodies against multiple FMD serotype infections. In comparison with the commercial PrioCHECK FMDV NSP-FMD test, there was a strong concordance and high correlation and agreement in the performance of the two tests. This new developed Nanobody based FMD 3ABC competitive ELISA could clearly benefit routine disease diagnosis, the establishment of disease-free zones, and the improvement of FMD management and control in endemically complex environments, such as those found in Africa.

## Introduction

Foot-and-mouth disease (FMD), is a highly contagious disease caused by FMD virus (FMDV), which is responsible for significant economic losses worldwide (1–3). FMDV is classified within the genus of Aphthovirus a member of the Picornaviridae family (4, 5). The genome consists of a single-stranded RNA, ~8 kb in length, which encodes four structural proteins (SPs, VP1, VP2, VP3, and VP4) and a total of ten mature non-structural proteins (NSPs; Lpro, 2A, 2B, 2C, 3A, 3B1-3, 3Cpro, 3Dpol (6, 7). The FMDV exists in the form of seven serologically and genetically distinguishable serotypes named A, O, C, Asia I, and South African Territories (SAT1, SAT2, and SAT3), with multiple subtypes within each serotype (8–10). Studies on outbreaks incidences showed that six of the seven serotypes of FMDV (O, A, C, SAT1, SAT2, and SAT3) have occurred in Africa (11, 12), and that currently, the predominant serotypes in Uganda are serotypes O, SAT1, and SAT2 (12–14).

Global FMD control strategy includes reliable and effective surveillance and is supported by competent laboratory diagnostic services (9, 15). Such diagnosis is typically carried out by the combination of virus isolation, serological tests, and nucleic acid recognition methods (9, 16). Serological tests are an essential component in the diagnosis algorithm of FMD because it is required for animal's import/export certification, as well as to determine the “free-from-infection” animal state and demonstrate vaccine efficacy (17). In this regard, the detection of antibodies to viral non-structural proteins, NSPs, is considered as one of the most important indicators of infection, irrespective of vaccination status (18), and is routinely performed in FMD free and endemic countries where vaccination is used (19).

Out of the different NSPs studied, the 3ABC polyprotein was found to be the most reliable single indicator of infection (20). Currently, most detection assays of antibodies to NSPs are based on recombinantly expressed 3ABC target antigen (21–26), and several 3ABC commercial tests (kits) are available today (17, 27). Although used worldwide, these tests vary in sensitivity and specificity and are expensive for developing countries (17, 27, 28).

Camilidae such as camels, llamas, and alpacas have a humoral immune response that has evolved into heavy-chain-only antibodies (29, 30). Unlike conventional IgGs, the antigen-binding fragment of these heavy chain antibodies consists of one single variable domain referred to as VHH or Nanobody (Nb) (31, 32). Nbs are typically procured by cloning their genetic repertoire from B cells circulating in the blood of an immunized animal, constructing a cDNA library and panning by phage display (31, 32). The Nb is one of the smallest known antigen-binding antibody fragments. Their reduced size, improved solubility, high stability, and antigen affinity makes them a great new generation of detection component for diagnostic applications (30, 33–35).

This study describes the development and validation of a new Nb-based FMD 3ABC competitive ELISA for the detection of anti-FMDV NSP antibodies in cattle serum in Uganda. The assay demonstrated high sensitivity and specificity to identify NSP antibodies of several FMD serotype infections with, effective and robust performance, and potentially low-cost production. This unique, tailor-made assay could clearly benefit routine disease diagnosis, the establishment of disease-free zones, and the improvement of FMD management and control in endemically complex environments, such as those found in Africa.

## Materials and methods

### Construction and expression of FMD 3ABC recombinant protein

FMDV 3ABC gene of serotype O (O1/Israel/99, GenBank: AF189157.1) containing inactivated 3Cpro protease (26) was codon optimized for expression in *E. coli* and synthesized commercially in the pJ411 expression vector (DNA2.0). In addition, a six-histidine sequence was added to the 5' end of the gene to generate a six-His (6xHis)-tagged protein. The cDNA construct was transformed into competent *E. coli* BL21(DE3) (Stratagene) and plated onto LB agar containing 25 μg/ml of Kanamycin (LB-Kan). A single colony of the transformed *E. coli* was inoculated into 10 ml of LB-Kan broth and cultured at 37°C overnight (ON) with vigorous shaking at 225 rpm. The ON culture was diluted 1:100 into LB-Kan and grown at 37°C with vigorous shaking until the optical density at 600 nm (OD600) reached 0.6–0.8. Then, the culture was supplemented with 1 mM isopropyl-β-D-thiogalactopyranoside (IPTG) and incubated at 37°C for an additional 4 h. Following incubation, cells were harvested by centrifugation at 6000 rpm for 20 min and frozen at −80°C until further use.

### Purification of FMD 3ABC recombinant protein

The *E. coli* pellet of the cells, containing FMDV 3ABC protein, was resuspended in a lysis buffer [50 mM NaH_2_PO_4_, 300 mM NaCl, 5 mM β- mercaptoethanol (β-Me) and 10 mM imidazole, pH 8.0], and 1 mg/ml Lysozyme, 3 U/ml Benzonase Nuclease, and protease inhibitor cocktail (Sigma-Aldrich) at 1:100 dilution was added to the lysis buffer. After 30 min incubation on ice, the bacterial cell wall was disrupted by ultra-sonication for 1 min at 80 % amplitude (repeated 3 times), on ice. Following the sonication, broken cells were centrifuged at 10,000 × g for 60 min at 4°C to separate the soluble and insoluble proteins fraction. FMDV 3ABC recombinant protein was purified from insoluble fraction and/or inclusion bodies. The insoluble fraction was washed with lysis buffer containing 1 % Triton X-100 followed by two washes with lysis buffer without Triton X-100. The insoluble material was dissolved in the denaturing solubilization buffer (50 mM NaH_2_PO_4_, [pH 8.0], 300 mM NaCl, 8.0 M Urea, and 1 mM DTT) and mixed on a platform shaker for about 1 h at Room Temperature (RT). Then, the mixture was centrifuged at 10,000 × g for 30 min at 4°C. The supernatant containing solubilized FMDV 3ABC was collected and loaded onto Ni-NTA resin (QIAGEN) which was pre-equilibrated with solubilization buffer. The protein was eluted from the column with solubilization buffer containing 0.25 M imidazole. Next, FMDV 3ABC protein was refolded by dilution to a uniform concentration of 0.7 mg/ml and dialyzed against refolding buffer containing 50 mM NaH_2_PO_4_, [pH 8.0], 150 mM NaCl, 8.0 M Urea, 3 mM reduced Glutathione, 0.3 mM oxidized Glutathione for 4 h at 4°C. Afterward, another dialysis step was performed against a buffer containing 50 mM NaH_2_PO_4_, [pH 7.5] 150 mM NaCl, 3 mM reduced Gluthatione, 0.3 mM oxidized Glutathione and 3.0 M urea, pH 8.0) at 4°C ON. The following day an additional dialysis step was performed for 4 h at 4°C against the same buffer with Urea concentration of 1.5 M. Final dialysis step was performed for 4 h at 4°C against a buffer containing 20 mM NaH_2_PO_4_, 150 mM NaCl, and 5 % v/v glycerol. The dialyzed refolded FMDV 3ABC protein was then concentrated using a Centricon 30 kDa cutoff, Millipore (MERCK).

The purity and integrity of FMDV-3ABC protein was assessed by SDS-PAGE and Western Blot (WB) as reported elsewhere (26). For the detection of the 3ABC protein an anti- FMDV-3ABC camel serum and a commercially anti-6xHis antibody (Sigma) were used as positive controls while serum from non-inoculated camel served as negative control.

### Ethical statement

All animal experiments were performed according to Directive 2010/63/EU of the European Parliament for the protection of animals used for scientific purposes and approved by the Ethical Committee for Animal Experiments of the Israel (clearance numbers 11-220-6 and 13-220-3).

### *Camelus dromedarius* immunization

A healthy camel was immunized with FMDV 3ABC protein or with four commercially synthesized (Sigma) peptides of 14 to 21 amino acids (aa) long, which represent conserved sequence motifs derived from FMDV 3ABC protein of all seven serotypes (36). All peptides were conjugated via their N terminal Cysteine to keyhole limpet hemocyanin (KLH) or to Bovine Serum Albumin (BSA; Sigma). The aa sequence of peptides used during immunization included; peptide 1A, CISIPSQKSVLYFLIEKGQHEA, derived from FMD 3A protein, peptide 1B, CGPYEGPVKKPVALKVKAK, derived from FMD 3B protein, and peptides 1C, CRVFEFEIKVKGQDMLSDAAL, and 2C, CMDGDTMPGLFAYRA, derived from FMDV 3C protein. During immunization, the camel was injected seven-times, once every 2 weeks, with FMDV antigen dissolved in PBS and mixed with an equal volume of Freund's incomplete adjuvant (Sigma). The first three injections included 1 mg/injection of the purified FMDV 3ABC protein. As from the fourth injection, the camel was injected with a mixture of 0.5 mg of FMDV 3ABC protein and the four different KLH- conjugated peptides, 100 μg/each. After seven injections, peripheral blood lymphocytes (PBLs) from 100 ml of the blood of the immunized camel were isolated by density gradient using HISTOPAQUE-1077 (Sigma) and used to construct the Nb library. All camel experiments were performed according to guidelines approved by the Israel Ethic Committee.

### Generation of phage-display library and selection of anti-FMDV-3ABC Nbs

The generation of anti-FMDV 3ABC Nb phage-display library was performed as previously reported (37). Briefly, PBL were purified from immunized camel by density centrifugation using Histopaque-1077 (Sigma-Aldrich). RNA was extracted using TRIzol reagent (Ambion) according to the manufacturer's instruction, and total cDNA was generated using SuperScript FIRST-Strand Synthesis System (Invitrogen) according to manufacturer's instruction. Total cDNA encoding all variable domains of both, conventional and heavy-chain-only antibodies was amplified by PCR using the primers CALL001 (5′-GTCCTGGCTGCTCTTCTACAAGG-3) and CALL002 (5′ GGTACGTGCTGTTGAACTGTTCC-3′). The shortest PCR amplicon (0.7 kb), comprising the variable domains that originate from heavy-chain only encoding mRNA, is purified from a preparative agarose gel and used for a nested PCR using the primers A6E (5′-GATGTGCAGCTGCAGGAGTCTGGRGGAGG-3′) and PMCF (5′-CTAGT GCGGCCGCTGAGGAGACGGTGACCTGGGT-3′). Afterward, PCR products and pMECS phagemid, were digested with PstI and NotI restriction enzymes (Roche). The pool of amplified Nb DNA fragments ligated in the phage-display vector pMECS was transformed into *E. coli* TG1 electrocompetent cells to generate a library of 1.0 × 10^7^ transformants. Next, Nbs were phage-displayed as previously described (37), and bio-panning procedures were performed against the FMDV 3ABC protein or the mixture of the four synthetic peptides, which were BSA conjugated for this step. Positive phage colonies were recovered by alkaline elution and reamplified for further use in a second and third round of bio-panning. After three enrichment rounds, ~100 colonies were randomly picked, and Nanobodies in the periplasmic extracts (PE) were screened against FMDV 3ABC protein and/or peptides for specific binders by ELISA as previously reported (37) with minor modifications. Briefly, cells containing Nbs were disrupted by osmotic shock, centrifuged and Nbs residing in the supernatant were then collected and incubated for 1 h at RT in microtiter plates (Nunc) precoated with 100 μl/well of 1 μg/ml FMDV-3ABC protein or individual synthetic peptides and incubated. All following assay procedures were performed as previously described (37). Colonies were considered positive when the ratio of OD405 nm between the test and control wells (non-coated well) was ≥ 3. Sequencing of positive-scoring constructs were then determined by an automated DNA sequencer (ABI Prism 3100 genetic analyzer; Applied Biosystems, Foster City, CA, USA), and Nb sequences were aligned.

### Purification of selected anti-FMD Nbs

Selected anti-FMDV 3ABC Nbs DNA fragments, fused to an HA tag and 6 x His tag at their C-termini in the pMECS vector, were transformed into *E. coli* WK6 and secreted into the periplasm. After ON bacterial induction at 28°C with 1 mM IPTG, periplasmic extracts containing the soluble anti-FMDV 3ABC Nbs were obtained by osmotic shock as previously reported (37). Nbs were then purified using immobilized metal affinity chromatography (IMAC) on Ni-NTA resin (Sigma-Aldrich, St. Louis, MO, USA) and gel filtration on Superdex 75 HR 16/60 (Pharmacia, Gaithersburg, MD, USA) in PBS. The concentration of the Nbs was determined by the OD_280nm_ measurement and using the individual theoretical extinction coefficients as calculated with the EXPASy - ProtParam webtool. Nbs were then aliquoted and stored at −80°C until further use.

### Sera samples

A total of 415 serum samples were collected from infected, non-infected and randomly recovered cattle herds obtained from the Uganda Virus Research Institute (UVRI), Entebbe, and Makerere University, Kampala, Uganda (Frank et al., under review). Sera were collected in Uganda in 2014 and 2015 during FMD outbreaks and in the following years during 2015 to 2017 from various cattle herds from previously infected districts as part of a national surveillance program. In addition, 216 serum samples collected from naïve and vaccinated calves were also obtained from the Kimron Veterinary Institute (KVI), Beit Dagan, Israel. A detailed description of all samples that were used during the study is presented in Table 1. All serum samples used during this work followed the procedures prescribed and approved by the Uganda institutional animal ethics committee.

**Table 1:** Sera sample bank.

**Sample details**	**Sample size**	**Collection Location**	**Collection year**
Naïve	72	Israel	2017
Vaccinated	144		2017
Non-infected	100	Uganda	
FMD Infected	150		
“O” strain	50		2014–2015
“SAT1” strain	50		
“SAT2” strain	50		
Randomly field collected	165		2016–2017
Total	631		

### Characterization affinity of Nbs to FMDV 3ABC recombinant protein

#### Surface plasmon resonance assay

The affinity of selected anti-FMDV 3ABC Nbs was measured using Surface Plasmon Resonance Assay (SPR) analysis as previously reported (37). The SPR binding studies were performed using a Biacore T200 instrument. A CM5 sensor chip (GE Healthcare) was coupled with 5 μg/ml FMDV 3ABC protein in 10 mM sodium acetate pH 5.5, using the amino coupling chemistry (NHS/EDC; N hydroxysuccinimide N-ethyl-N'- (dimethylaminopropyl carbodiimide), as recommended by the manufacturer. The final change in response units (RU) was 840 RU. For affinity measurements, different concentrations ranging from 500 nM to 1.95 nM of purified Nbs were injected over the sensor chip at a flow rate of 30 μl/min in HEPES-buffered saline (HBS; 10 mM HEPES, pH 7.5, 150 mM NaCl, 3.5 mM EDTA and 0.005 % (v/v) Tween 20) running buffer. The contact time was 120 s followed by a dissociation time of 600 s. Regeneration was performed for 60 s with 100 mM Glycine pH 2.0 followed by a stabilization time of 600 s. Kinetic parameters were evaluated with the help of the BIA evaluation T200 software (Biacore) assuming a 1:1 Langmuir binding, with drift.

#### Indirect ELISA

The ELISA procedure was based on a previously described method (23, 24) with minor modifications. Briefly, Maxisorb ELISA plates (Nunc) were coated at 4°C for 16 h (ON) with 100 μl/well of FMDV 3ABC protein at a concentration of 60 ng/ml suspended in PBS. Following incubation plates were washed three times with washing buffer [PBS containing 0.05 % Tween 20 (PBS-T)] and then blocked for 1 h at RT with 5 % skimmed milk (Sigma). The same washing procedure was performed after each incubation step. Next, serial dilutions of tested Nbs in concentrations from 4 μg/ml to 60 ng/ml were added at a volume of 100 μl/well and incubated for 1 h at RT. Following incubation plates were washed and 100 μl/well of 1 μg/ml anti-HA (Sigma) was added and incubated for 1 h at RT. Afterward, plates were washed and 100 μl/well of 1 μg/ml HRP-conjugated anti-mouse IgG antibody (Sigma) was added and incubated for an additional 1 h at RT. Finally, plates were washed four times and 90 μl/well of TMB One solution (SouthernBiotech) was added to develop the color reaction. The color reaction was stopped after 15 min with 1 M Sulfuric acid and readouts were obtained with absorbance read at 470 nm using a standard luminometer (Thermolabsystems-Luminoskan Ascent).

### Nb-based 3ABC competitive ELISA

The levels of circulating antibodies to FMDV 3ABC protein were determined by a novel Nb-based competitive NSP ELISA. In principle, immunoassay plates (Maxisorp, Nunc) were coated ON at 4°C with FMDV 3ABC protein at a concentration of 60 ng/ml in PBS. Following incubation plates were washed three times in wash buffer (PBS containing 0.1 % Tween-20) and blocked 1 h at RT with 5 % skimmed milk. The same washing procedure was performed after each incubation step. After plates were washed, serum samples diluted 1:25 in diluent buffer (PBS containing 0.1 % Tween-20 and 1 % skimmed milk) were dispensed at a volume of 100 μl/well in duplicate and incubated ON at 4°C. Following incubation, plates were washed, and 100 μl/well of 0.25 μg/ml of the incubated Nb in dilution buffer at a volume of 100 μl/well was added and incubated for 1 h at RT. Subsequently, plates were washed, and a commercial monoclonal anti-HA antibody (Sigma-Aldrich) diluted 1:2000 in dilution buffer was added into the wells and incubated for 1 h at RT. Then after washing the plates, 100 μl/well of 1:2000 rabbit anti-mouse IgG conjugated to HRP was added and incubated for additional 1 h at RT. Finally, plates were washed four times and 90 μl/well of TMB One solution (SouthernBiotech) was added to develop the color reaction. The color reaction was stopped after 15 min with 1 M Sulfuric acid and readouts were obtained with absorbance read at 470 nm using a standard luminometer (Thermolabsystems-Luminoskan Ascent).

#### Nb based 3ABC competitive ELISA cutoff value

The cutoff value was determined with a control set of 150 negative cattle sera collected from uninfected animals in Uganda (Frank et al., under review) and using 10-fold stratified cross-validation analysis (38). Percent Inhibition (PI) values were calculated for each serum tested using the formula: 100–(OD tested sample/ OD negative control) × 100. The signal of the negative control is obtained by adding no serum only 0.25 μg/ml of Nb at a volume of 100 μl/well. Samples showing a PI value above 51 % were considered “positive”; and those below 51 %, “negative.”

#### Nb-based 3ABC competitive ELISA analytical sensitivity

The analytical sensitivity of the Nb-based NSP competitive ELISA was assessed by determining the endpoint dilution of a positive control serum using a 2-fold dilution series from 1:5 to 1:320. The endpoint was the dilution at which the value for the positive test sample was below the cutoff value and could not be discriminated from that of the negative control.

#### Nb-based 3ABC competitive ELISA diagnostic sensitivity (DSe) and specificity (DSp)

The Nb-based NSP competitive ELISA was evaluated with diagnostic performance parameters using sera (*n* = 466) from cattle with a known FMDV infection, and non-infected or unexposed cattle sera (Frank et al., under review). Samples (*n* = 316) collected from cattle with no previous history of exposure to FMDV or vaccination, which tested negative in the PrioHECK FMDV NSP test were used to estimate the relative DSp of the Nb-based 3ABC competitive ELISA. Of these, 72 were from naïve and 144 form vaccinated calves collected in Israel. DSe was calculated using a total of 150 sera samples from post-outbreak sera from naturally infected animal's cattle collected in Uganda and confirmed as having been infected using the PrioCHECK FMDV NSP test.

#### Nb-based 3ABC competitive ELISA comparison with prioCHECK FMDV NSP test

PrioCHECK FMDV NSP test (Prionics Lelystad, The Netherlands) was carried out following manufacturer's instructions. After color development was stopped PI values were calculated. Samples showing a PI value above 50 % were considered “positive” for FMDV NSP antibodies; and those below 50 %, “negative.”

### Statistical analysis

Statistical analyses were performed using GraphPad Prism software 6.01 (GraphPad Software, Inc. LA Jolla, CA, USA). Differences in values between study groups were assessed by analysis of variants (ANOVA) and Wilcoxon rank sum test; *p*-values represent 2-sided *p*-values, and *p*-values < 0.05 were considered statistically significant. For statistical comparison, the correlation, and agreement analysis between the Nb-based 3ABC competitive ELISA and PrioCHCK FMDV NSP test was done by calculating the kappa coefficient, and preforming Bland-Altman analysis (GraphPad), respectively, for the results obtained in both tests across all categories of sera.

## Results

### Construction of FMD 3ABC recombinant protein and peptides

A recombinant 3ABC protein was used for the detection of specific FMDV NSP antibodies. The construction and cloning of the FMDV 3ABC recombinant viral gene (serotype O1, GenBank: AF189157.1) included an inactivated 3Cpro/mut and a 6 × His tag marker. In addition, four peptides derived from FMDV genes 3A, 3B, and 3Cpro were also synthesized (one from 3A and 3B and two from 3C referred to as 3C1 and 3C2). These specific peptides were selected based on their amino acid sequence conservation among the seven serotypes of FMDV and therefore have the potential to interact with antibodies against NSP of multiple viral serotype strains. WB analysis was performed for the detection of FMDV 3ABC protein, using an FMDV-3ABC immunized camel serum, vs. a non-immunized camel serum as negative control. Since FMDV-3ABC recombinant was recombinantly expressed as a His-tagged protein, an anti-His monoclonal antibody was also used as a second positive control. The results presented in Figure S1 shows a specific distinct band, corresponding to a molecular weight of ~50,000 kd representing FMDV 3ABC recombinant protein. This distinctive band was only detected when blotted against immunized camel serum or with an anti-His tag antibody control (Figure S1A). As expected non-immunized serum showed no immunoreactivity toward FMDV 3ABC protein. Additional immunoreactivity specificity was also validated by using the FMDV 3D recombinant protein as a negative antigen control. The results of 3ABC immunized camel sera blotted against these two recombinant viral proteins showed a distinctive band only against FMDV 3ABC recombinant protein blot (Figure S1B). Coomassie blue staining result is also presented (Figure S1C).

### Selection of anti-FMDV 3ABC protein nanobodies

**Figure 1:**
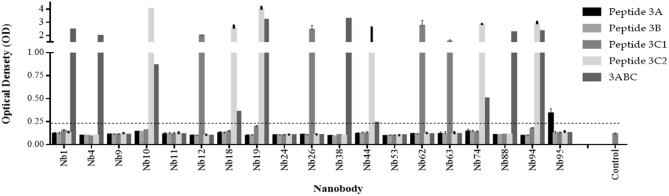
Selection of Nanobodies (Nbs) from an immune phage-displayed Nb library against FMDV 3ABC protein. Periplasmic Extract (PE) ELISA results demonstrating 19 Nb colonies analyzed for the immunorecognition to FMDV 3ABC protein and peptides (3A, 3B, 3C1, and 3C2). Luria-Bertani (LB) broth medium was used as negative control. Assay cutoff is indicated by dash line.

The Nbs against FMDV 3ABC recombinant protein were identified and isolated from an immune phage-displayed Nb library as schematically shown in Figure S2. A total of more than 100 Nbs were isolated and tested, against FMDV 3ABC recombinant protein and peptides (3A, 3B, 3C1, and 3C2). Screening results after panning, partly presented in Figure 1, reveal five classes of Nbs with distinctive immunorecognition profile to FMDV protein and peptides. Most of the isolated Nbs were able to demonstrate positive immunorecognition of either a single FMDV peptide or the protein. Out of a total of 100 Nbs tested, two Nbs, (i.e., Nb19 and Nb94) showed the capacity to strongly recognize 3ABC recombinant protein as well as an additional viral peptide 3C2. These two Nbs, along with four other Nbs (i.e., Nb1, Nb4, Nb38, and Nb88) that demonstrated high immunoreactivity solely to FMDV 3ABC recombinant protein, were selected for further evaluation. Additionally, Nb9, which showed low immunoreactivity to FMDV 3ABC recombinant protein or peptides, was also used as a negative control (Figure 1).

**Figure 2:**
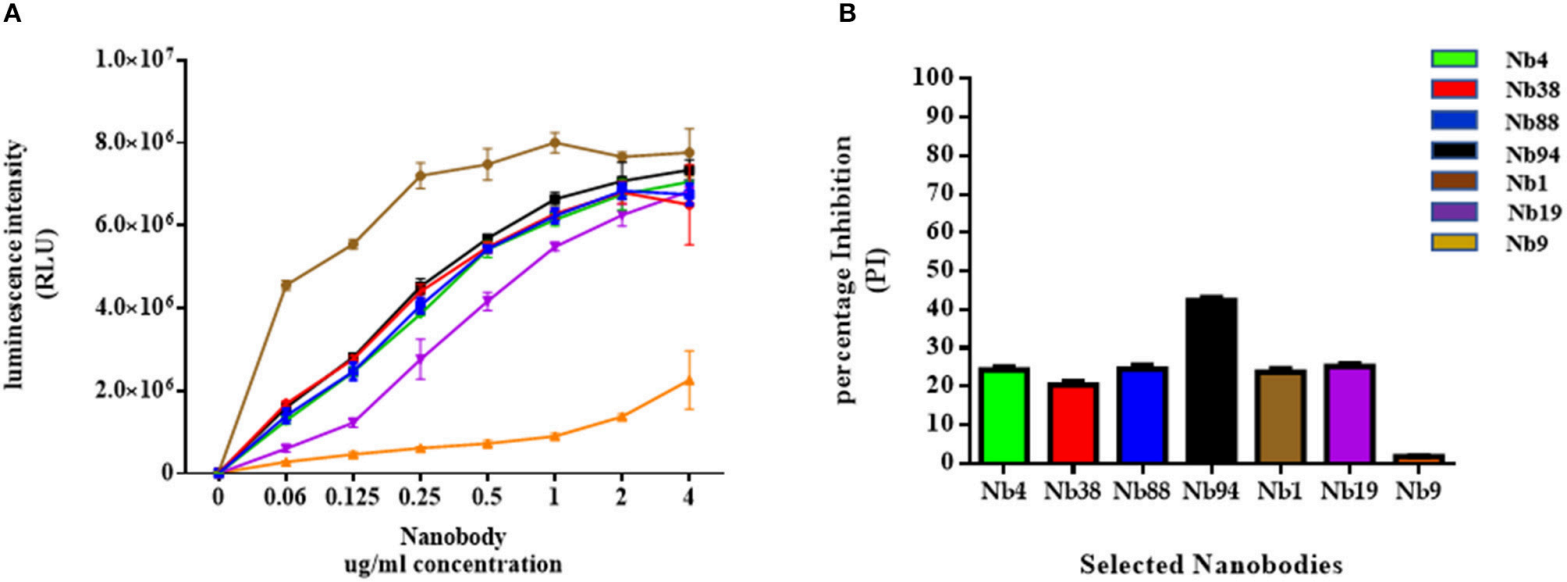
Binding affinity analysis of selected Nanobodies (Nbs) against FMDV 3ABC protein. **(A)** The binding affinity results of six anti-FMDV 3ABC Nbs, evaluated by indirect ELISA. During experiments Nb9 was used as a negative control. The results are presented in Relative Light Units (RLU). **(B)**. The immunorecognition performance of six selected anti-FMDV 3ABC Nbs in a competitive ELISA format using a set of 8 infected and noninfected control samples. Mean percentage of inhibition (PI) for each Nb was calculated using the formula: 100–(X Aver infected/X Aver noninfected) × 100.

The selected Nbs were then evaluated for their binding affinity to FMDV 3ABC recombinant protein using SPR analysis and indirect ELISA. The SPR results presented in Table S1 show that five out of six Nbs tested had high binding affinity to FMDV recombinant 3ABC protein, with KD-values ranging from 1.67 to 9.37 10^−8^ M (Table S1). The indirect ELISA data presented in Figure 2A yielded similar binding affinity as the SPR analysis, demonstrating all Nb tested to have high immunoreactivity to FMDV 3ABC recombinant protein. As expected Nb9 showed low binding affinity and immunoreactivity capacity in both methods (Table S1 and Figure 2A). Additional assessment of Nbs performance was carried out using the preliminary format of the competitive ELISA. The results presented in Figure 2B revealed that out of the six Nbs, Nb94 demonstrated the highest PI of 40 %, compared to ~20 % for the other Nbs tested. Based on the overall performance, Nb94 was selected for the construction of the in-house Nb-based 3ABC competitive ELISA.

### The construction of the in-house Nb-based FMD 3ABC competitive ELISA

**Figure 3:**
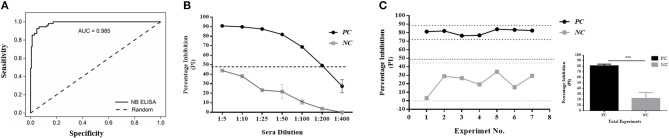
Diagnostics performance of the Nanobody (Nb94) based 3ABC competitive ELISA. **(A)** Receiver-Operating Characteristic (ROC) analysis results from a total of 222 cattle sera. Dotted lines represent 95 % confidence interval. **(B)**. Lower limit of detection (LLD) using positive (infected sera) and negative (noninfected sera) control serum. Cutoff assay is indicated by dash line. **(C)** Intra assay repeatability performance was assessed using a set of positive and negative internal control sera tested in the assays in different days and by different operators. Lower and upper limits (2 × STDV) are indicated by dot lines. Cutoff assay is indicated by dash line. The total average and STDV of positive and negative internal control sera across the different experiments is also presented. Statistical analysis: ^*^P < 0.05, ^**^P < 0.01, ^***^P < 0.001.

Using Nb94 as a competitive component, an in-house 3ABC ELISA for the detection of FMD NSP antibodies in cattle serum was developed and validated. The development of the assay included the assessment of various parameters such as the antigen and Nb94 concentration, sera dilution, incubation times, and temperature conditions (represented in Figure S3). A schematic presentation of the assay configuration is shown in Figure S4. In addition, an evaluation was performed for the analytical performance of the assay, including diagnostically sensitivity and specificity, lower limit of detection, and repeatability (Figure 3). The results presented in Figure 3A show the assay to be highly predictable with an AUC (Under the Curve) of 0.985 as determined by Receiver-Operating Characteristic analysis (ROC) using a total of 222 sera (from infected, non-infected, and naïve animals). The Nb-based 3ABC competitive ELISA demonstrated high analytical sensitivity and specificity of 94 % (95 % CI: 88.9–97.2) and 97.67 % (95 % CI: 94.15–99.36) respectively. Lower limit of detection calculated using a set of positive (infected sera sample) and negative (non-infected sera sample) control serum, and presented in Figure 3B, demonstrated clear discrimination between seropositive and negative controls using dilutions in the range of 1:5 to 1:200, with positive control yielding high PI values (≈ 90 %) down to a 1:50 dilution, after which the PI values gradually decreased. Inhibition was still seen at a dilution of 1:200. Overall the assay was highly repeatable, as determined by the set of the positive and negative controls tested on different days and by different operators (Figure 3C).

### Sera screening

#### Infected, non-infected, naïve, and vaccinated samples

**Figure 4:**
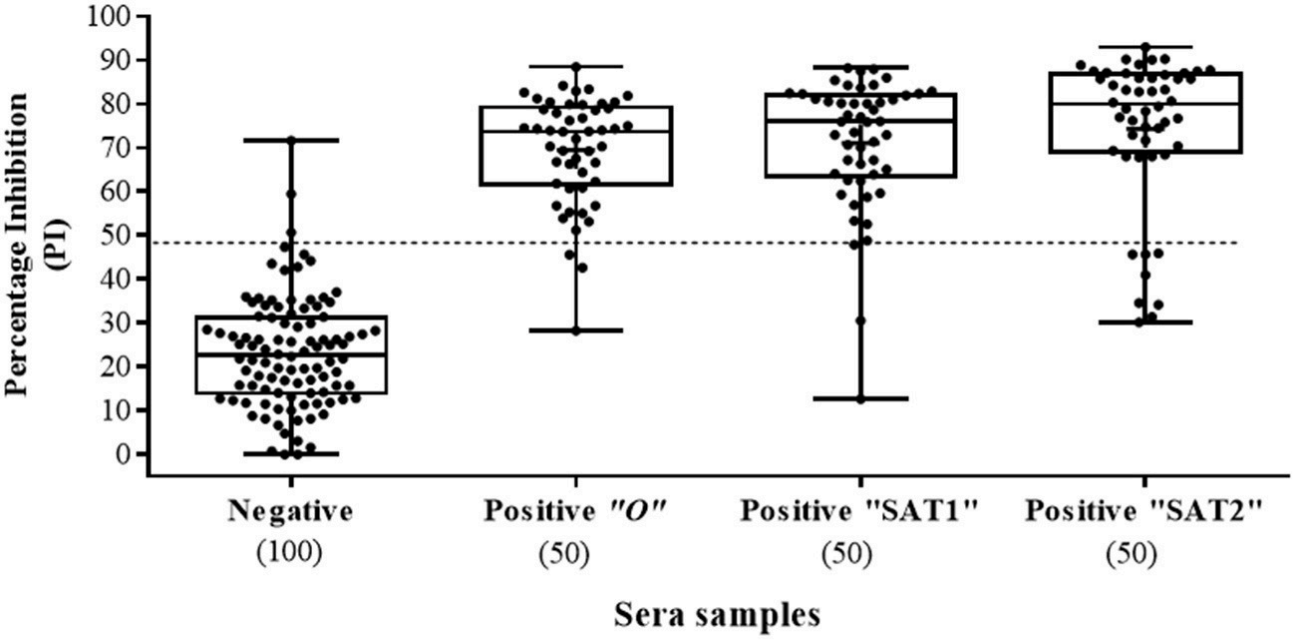
Sera screening analysis of infected and noninfected cattle samples using the Nanobody (Nb94) based 3ABC competitive ELISA. Box plot representing 100 noninfected (negative) and 150 infected (confirmed by reverse transcription PCR or virus isolation) (positive) sera collected in Uganda. FMDV serotype of infected cattle, included serotype O, SAT1 and SAT2 (50 samples for each). Cutoff assay is indicated by dash line. Numbers in brackets indicate the total number of tested sera.

**Figure 5:**
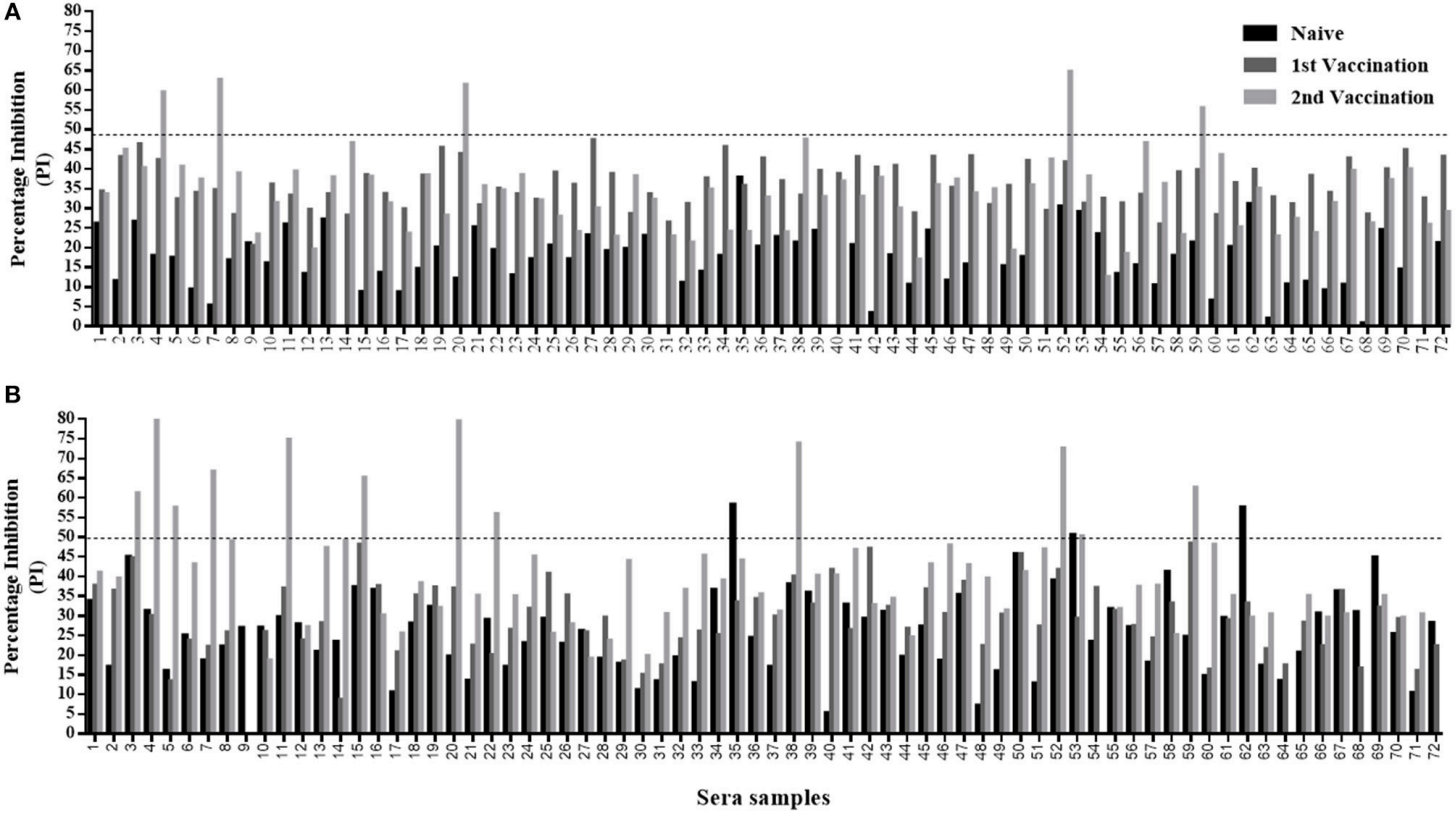
Sera screening analysis of naïve and vaccinated calves' samples using the Nanobody (Nb94) based 3ABC competitive ELISA and PrioCHECK NSP test. A total of 72 calves were serially sampled three times; before first vaccination (naïve state), after first vaccination, and after second vaccination. The prevalence of FMDV NSP antibodies were determined by the Nb (Nb94) based 3ABC competitive ELISA **(A)** and PrioCHECK NSP test **(B)**. Cutoff for each assay is indicated by dash line.

**Figure 6:**
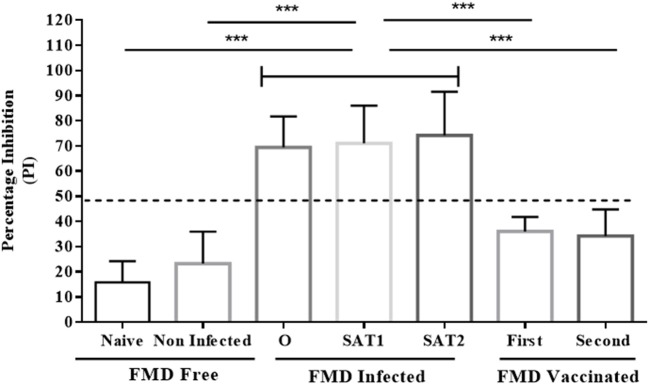
Comparison analysis of serum samples tested by the Nanobody (Nb94) based 3ABC competitive ELISA. Serum collected from cattle with different FMD status were analyzed for NSP antibodies presence by the Nb (Nb94) based 3ABC competitive ELISA. These included free (naïve and noninfected), infected (FMDV serotype O, SAT1 and SAT2) and vaccinated (first and second vaccination). The mean results of percentage of inhibition (PI) is presented. Cutoff assay is indicated by dash line. Statistical analysis: ^*^P < 0.05, ^**^P < 0.01, ^***^P < 0.001.

A total of 250 serum samples collected in 2014-2015 from infected and non-infected cattle herds in Uganda were delivered to UVRI. Out of these, 150 and 100 samples were previously tested using commercially-available PrioCHECK NSP ELISA (39) and classified as positive or negative for the presence of antibodies against FMDV NSP, respectively. Out of the 150 serum samples three groups of 50 each, were obtained from animals from which FMDV serotype O, serotype SAT1, and serotype SAT2 were isolated from esopharingeal fluids (probang) samples (Frank et al., under review; (40)). A comprehensive screening analysis of these samples was performed by the Nb-based NSP competitive ELISA. The results presented in Table 2 and Figure 4 showed that 94 % of all infected samples (141 out of 150) were positive for NSP antibodies, where 98 % were diagnosed positive for serotype O samples (49 out of 50), 96 % for serotype SAT1 (48 out of 50), and 84 % for serotype SAT2 (42 out of 50). Screening results of non-infected serum samples demonstrated high specificity of 96 %, with 96 out of the 100 samples tested were negative for NSP antibodies presence (Table 2 and Figure 4). In addition, a total of 216 serum samples were obtained from KVI, Bet Dagan, Israel. These samples were collected from 72 calves at three different time points representing different FMD vaccination status: naive uninfected and unvaccinated, and those that received first vaccination and second vaccination. The results presented in Table 2 and Figure 5A showed the Nb-based 3ABC competitive ELISA to have 100 % specificity for the naïve group (72 out of 72 negatives), 99 % for calves after first vaccination (71 out of 72 negative), and 93 % for calves after two sets of vaccination (67 out of 72 negative). Comparison of the different sample groups presented in Figure 6 demonstrated significant discrimination (*P* < 0.001) between FMD infected samples to FMD free, and vaccinated, with mean PI of 70 compared to 20 and 35 %, respectively.

**Table 2:** Performance of Nanobody (Nb94) based 3ABC competitive ELISA in comparison to FMDV PrioCHECK NSP test.

**Sample Category**	**Sample size**	**In-house 3ABC competitive ELISA**		**FMDV PrioCHECK NSP test**		**Concordance (%)**
		**Positive (%)**	**Negative (%)**	**Positive (%)**	**Negative (%)**	
Naïve	72	0/72 (0 %)	72/72 (100 %)	2/72 (3 %)	72/72 (97 %)	97 %
Non-infected	100	4/100 (4 %)	96/100 (96 %)	3/100 (3 %)	97/100 (97 %)	99 %
FMD Infected	150	141/150 (94 %)	9/150 (6 %)	148/150 (98 %)	2/150 (2 %)	95 %
“O” strain	50	49/50 (98 %)	1/50 (2 %)	50/50 (100 %)	0/50 (0 %)	99 %
“SAT1” strain	50	48/50 (96 %)	2/50 (4 %)	49/50 (98 %)	1/50 (2 %)	99 %
“SAT2” strain	50	42/50 (84 %)	8/50 (16 %)	49/50 (98 %)	1/50 (2 %)	88 %
Vaccinated	144	6/144 (4 %)	138/144 (96 %)	12/144 (8 %)	132/144 (92 %)	96 %
1st vaccination	72	1/72 (1 %)	71/72 (99 %)	0/72 (0 %)	72/72 (100 %)	99 %
2nd vaccination	72	5/72 (7 %)	67/72 (93 %)	12/72 (17 %)	60/72 (83 %)	90 %
Total	466					

#### Randomized cattle herds in uganda

**Figure 7:**
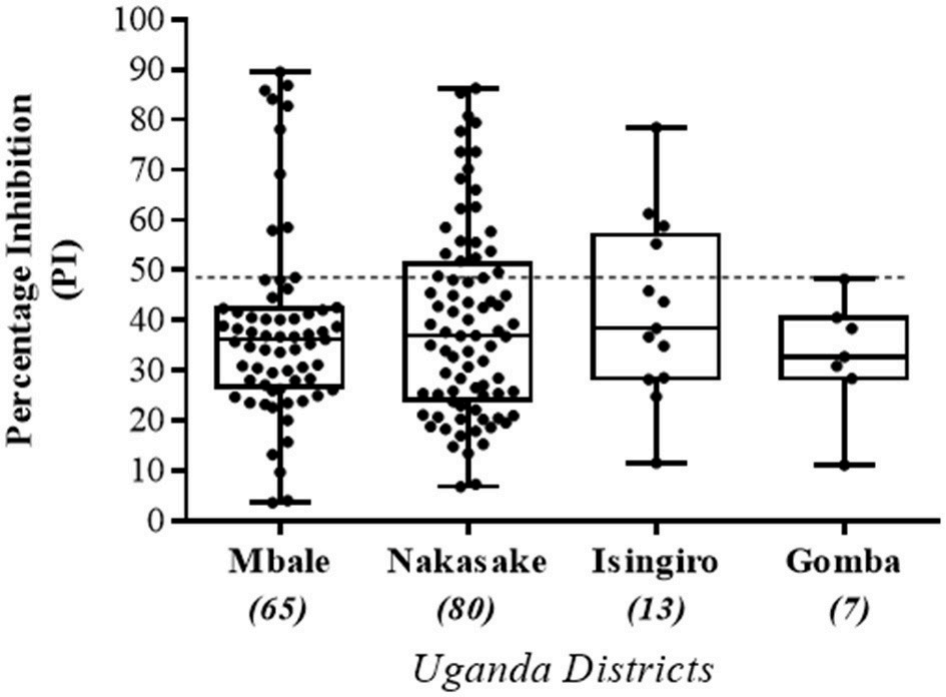
Sera screening of randomly collected field samples tested by the Nanobody (Nb94) based 3ABC competitive ELISA. A total of 165 cattle serums previously collected from four different districts (Mbale, Nakasake, Insingiro, and Gomba) in Uganda, by the Uganda Virus Research Institute (UVRI), were obtained and analyzed using the Nb (Nb94) based 3ABC competitive ELISA. Samples were collected as part of an FMD surveillance program for assessing the levels of NSP antibodies prevalence in the country. Cutoff assay is indicated by dash line. Numbers in brackets indicate the total number of sera tested in each district.

The UVRI health workers collected a total of 165 serum samples from four districts in Uganda during 2016-2017 as part of a sera surveillance study to assess the levels of FMD NSP antibodies in cattle herds. These samples were analyzed using the Nb-based 3ABC competitive ELISA. Out of the 165 samples, 80 samples were collected in Nakasake district, 65 in Mbale district, 13 in Isingiro district, and 7 in Gomba district (see Table 3). The results presented in Table 3 and Figure 7 showed that 21 out of 80 samples collected in Nakasake were defined positive for the presence of FMD NSP antibodies, 9 out of 65 in Mbale, 4 out of 13 in Isingiro, and 0 in Gomba. In total, a prevalence of 20 % of FMD NSP antibodies (34 out of 165), was found in these groups of randomly collected samples.

**Table 3:** Sera screening analysis, of randomly collected field samples, tested by the Nanobody (Nb94) based 3ABC competitive ELISA and FMDV PrioCHECK NSP test.

**Field collected samples**	**Sample size**	**In-house 3ABC competitive ELISA**		**FMDV PrioCHECK NSP test**		**Concordance (%)**
**Uganda district**		**Positive (%)**	**Negative (%)**	**Positive (%)**	**Negative (%)**	
Nakasake	80	21/80 (26 %)	59/80 (74 %)	20/80 (25 %)	60/80 (75 %)	99 %
Mable	65	9/65 (14 %)	56/65 (86 %)	15/65 (23 %)	50/65 (77 %)	90 %
Isingiro	13	4/13 (30 %)	9/13 (70 %)	5/13 (38 %)	8/13 (62 %)	92 %
Gomba	7	0/7 (0 %)	7/7 (100 %)	0/7 (0 %)	7/7 (100 %)	100 %
Total	165	34/165 (20 %)	131/165 (80 %)	40/165 (24 %)	125/165 (76 %)	96 %

### Diagnostic performance of the Nb-based 3ABC competitive ELISA compared to priocheck Nsp test

The Nb-based 3ABC competitive ELISA results were compared with a PrioCHECK FMDV NSP test. A total of 631 serum samples were tested and analyzed in both assays. These samples represented different FMD state, including naïve, non-infected, infected, vaccinated, and randomized field samples survey (Table 1). The results presented in Table 2 and Table 3 showed a strong correlation between the Nb-based 3ABC competitive ELISA and PrioCHECK FMDV NSP test. High concordance between the two assays was observed for the samples collected from naïve, non-infected, FMD serotype O and SAT1 infected, and first time vaccinated calves (97–99 %). Samples collected from animals infected with FMD serotype SAT2 and second time vaccinated animals showed a lower concordance of 88–90 % (Table 2). The comparison of the randomized field samples survey exhibited an accordance of 96 % between both assays (Table 3). Data analysis revealed 7 % (5/72) of animals that received two vaccinations were diagnosed positive for the presence of NSP antibodies when tested by the Nb-based 3ABC competitive ELISA, compared to 16.6 % detected in the PrioCHECK test (12/72) (Figures 5A,B, respectively). Similar prevalence levels were seen in both assays for samples from infected and randomly collected animals (98 % to 94 %, and 24 % to 20 %, respectively). Data analysis, estimated by calculating the kappa coefficient value (GraphPad software), demonstrated a strong correlation between both assays with a value of 0.875 and SE of 0.021 (95 % CI: 0.834 to 0.916) for all samples across all categories. Further data analysis using Bland-Altman method (GraphPad software) presented in Figure S5, also revealed high agreement between both assays with a Bias value of 0.92, SD of 1.36 and 95 % limits of agreement; −1.74 to 3.58.

## Discussion

FMD is an acute and highly contagious disease of cloven-hoofed animals, which can lead to devastating economic losses across many parts of the world (9, 41). Over the years, extensive efforts have been invested to improve the performance of diagnostic tests for FMD, resulting in the development of a wide range of ELISA tests to detect the presence of anti-NSP antibodies (22, 23, 25, 39, 42, 43). Although several 3ABC commercial tests (kits) are available, these tests are not ideal since they are extremely expensive and have raised concerns regarding sensitivity and specificity performance, especially in endemically complex surroundings (15, 44–46).

To address the environmental needs and to overcome the challenges of the endemically multiple settings, a novel Nb-based FMD 3ABC competitive ELISA was developed for the detection of antibodies against NSP in cattle sera in Uganda. The design of this in-house assay included the use of a high-affinity Nb (Nb94), which targets the FMDV 3ABC protein, and more specifically, a conserved region located within the FMDV 3Cpro protein. The selection of this Nb with an immunoreactivity profile to both the complete protein and a specific conserved region was proven to be highly critical, enabling the detection of multi-FMD serotype strains in a single assay configuration. The nature characteristics of Nbs, including their structural stability, solubility, scalable and straightforward production, their elevated thermostability, and long shelf-life (47) were the key reasons for using them as the competitive component in the development of the assay. Also, the use of Nbs provides a significant economic benefit. They potentially enable development of detection tools, with longer shelf-life and eliminates the need for temperature-controlled storage and supply chain, which are extremely important in changing surrounding such as presented in Africa. Also compared to mAbs, Nbs distinctive structural properties (devoid of a light chain, and only comprising a single, variable heavy chain domain) facilitate their construction, which results in lower production costs. They were shown to be efficiently expressed in economic production systems, such as bacteria and yeast, with high batch-to-batch consistency (48), which allow their production on a large scale and a short period. Considering the above, the use of Nbs in our competitive ELISA format provides the assay, if needed, the capacity for easy adjustment for specific detection of other FMDV serotypes based on 3ABC or any other proteins selected.

The new Nb-based competitive ELISA was designed for detection of antibodies against FMD NSP. This was done since NSP antibodies have been widely accepted as a reliable method for diagnosing the infection status of animal herds, regardless of vaccination status (17, 49–52). Furthermore, the presence of these antibodies provides critical input for the risk analysis in the assessment of FMD control management (44, 53), and it is currently the most sensitive tool to distinguish present from past infection with FMDV after a single time-point sampling (17).

The Nb-based 3ABC competitive ELISA was evaluated by screening a wide range of serum samples representing different FMD serotype infection and state. These serum samples were obtained from cattle herds in Uganda and Israel considered FMD free (naïve and clinically non-infected), FMD infected (serotype O, SAT1, and SAT2 infection), and FMD vaccinated (first or second vaccine admission). The analytical performance of the assay was assessed using ROC analysis, which demonstrated the assay high predictive strength, as well as its high diagnostic sensitivity and specificity. Strong repeatability and clear discrimination between infected animals, naive/non-infected, and vaccinated animals was also observed. The screening results demonstrated the Nb-based 3ABC competitive ELISA could successfully differentiate between infected and vaccinated animals (DIVA). This capability was highlighted by the high numbers of positive samples detected by the assay in the infected animal group (141 out of 150), compared to the low to non-positive samples determined in the vaccinated (1 out of 72 after first vaccination, and 5 out of 72 after second vaccination), and naive (0 out of 72) groups. The assay showed also the capacity to detect NSP antibodies in sera samples collected from cattle infected by three different FMD serotypes, with total sensitivity of 94 % (95 % CI: 88.9–97.2) and specificity of 97.67 % (95 % CI: 94.15–99.36), as determined by testing a set of naïve and non-infected samples.

The Nb-based 3ABC competitive ELISA performance was compared with the commercial PrioCHECK NSP ELISA test that is widely used in Uganda (17, 49). This comparison demonstrated high correlation and agreements between assays for all serum samples regardless of their FMD status. Interestingly, although both assays showed that calves exhibited an increase in their NSP antibody response after two shots of vaccines, the PrioCHECK NSP ELISA test has defined a double number of positive samples compared to the Nb-based 3ABC ELISA (12 and 5, respectively). Although limited by sample numbers, this result is consistent with previous reports showing that the specificity of the PrioCHECK NSP test dropped significantly after multiple doses of vaccination (17, 43, 54). In theory, the detection of antibodies against NSPs indicates infection rather than vaccination, however, in practice, antibodies against NSPs may also be provoked by trace amounts of NSPs present in commercial vaccines and multiple vaccinations (18, 20, 50, 55). Since under ideal conditions vaccinated animals should not elicit NSP antibodies, the lower positive NSP antibodies samples seen by the Nb-based 3ABC competitive ELISA could suggest a higher specificity of our assay compared to the PrioCHECK NSP test. To further validate assay performance, a small randomized trial consisting of serum samples collected from individual cattle field herds in different districts in Uganda were also analyzed. The results revealed a total prevalence level of 20 % for NSP antibodies by the Nb-based 3ABC competitive ELISA. Although this cohort represents limited sample numbers, the integrity of the assay performance is supported, by the PrioCHECK NSP test analysis that demonstrated a similar NSP antibody prevalence result of 25 %.

Today, most African countries are still poorly equipped to control FMD due to lack of infrastructure and financial resources (15, 45). FMD diagnosis in countries such as Uganda is mainly based on molecular diagnostic tests and serological assays such as NSP ELISA (39). Although molecular-based diagnostic tests have shown a higher analytical sensitivity compared to serological assays, these systems require sophisticated equipment and highly trained laboratory staff. Such limitations make them not practical for routine screening and confine their use to research institutions (45, 56, 57). As a result, the focal testing of FMD is carried out in regional and national reference labs and mainly relies on commercial NSP ELISA kits (39, 56). Considering the unique environmental and economic challenges, new tailored serological assays with high diagnostics performance, low production cost, and without the need for expensive laboratory equipment are clearly still needed for large-scale application in FMD control and surveillance.

In this study, we successfully developed and validated a DIVA Nb-based 3ABC competitive ELISA, for detection of NSP antibodies in cattle serum. Since every assay development has its own sets of merits and demerits (58), various parameters that extensively differ between non-endemic and endemic surroundings, such as those seen in Uganda, were taken under consideration during assay design. Further studies with large sample cohorts and across different animal species must still be carried out to validate the assay performance before regulatory authorities can adopt it for routine use. However, this tailor-made highly sensitive and specific NSP ELISA presented herein clearly demonstrates the potential to be used as an alternative/supplemental way for simple, low-cost and effective method for detection of FMD NSP antibodies, and to serve as a critical component in FMD regional control management and surveillance.

## Author contributions

Study experiments were designed by SG, AS, ElR, SM, VY, and LL. SG designed FMDV peptides and constructed FMDV 3ABC protein. SG, CV, and EmR selected, expressed, purified, and characterized the anti-FMD 3ABC nanobodies. FM and SO collected analyzed and provided FMD infected sera samples. Naive and vaccinated samples were collected analyzed and provided by NS. Randomized field samples were collected and process by IA and JL. SG, AS, PB, and SF-M developed and constructed the Nb-based 3ABC competitive ELISA. ELISA screening analysis was performed by SG, AS, and PB. PrioCHECK NSP test analysis was done by IA, SG, AS, and PB. The manuscript was written by AS and SG and edited by RM, LV-S, ElR, SM, CV, JL, VY, and LL.

### Conflict of interest statement

The authors declare that the research was conducted in the absence of any commercial or financial relationships that could be construed as a potential conflict of interest.
