# Supplementary material for: The Development and Validation of a Novel Nanobody-Based Competitive ELISA for the Detection of Foot and Mouth Disease 3ABC Antibodies in Cattle
Source: Front Vet Sci. 2018 Oct 12;5:250. doi: 10.3389/fvets.2018.00250 (PMC6194346; doi:10.3389/fvets.2018.00250)
Supplement: Supplementary file 1 [file Presentation_1.pptx]

## Slide 1
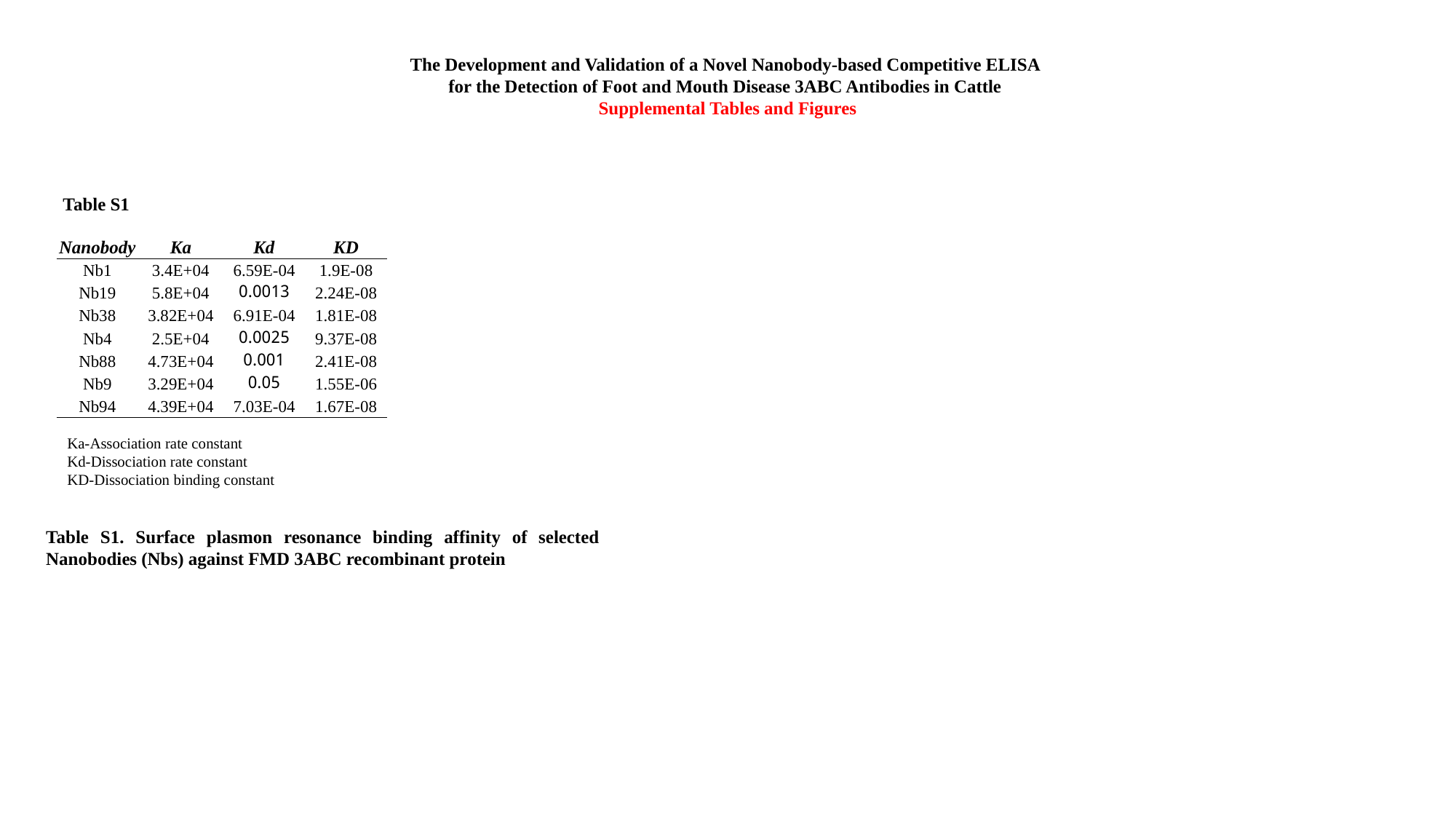

The Development and Validation of a Novel Nanobody-based Competitive ELISA
for the Detection of Foot and Mouth Disease 3ABC Antibodies in Cattle
Supplemental Tables and Figures
Table S1
| Nanobody | Ka | Kd | KD |
| --- | --- | --- | --- |
| Nb1 | 3.4E+04 | 6.59E-04 | 1.9E-08 |
| Nb19 | 5.8E+04 | 0.0013 | 2.24E-08 |
| Nb38 | 3.82E+04 | 6.91E-04 | 1.81E-08 |
| Nb4 | 2.5E+04 | 0.0025 | 9.37E-08 |
| Nb88 | 4.73E+04 | 0.001 | 2.41E-08 |
| Nb9 | 3.29E+04 | 0.05 | 1.55E-06 |
| Nb94 | 4.39E+04 | 7.03E-04 | 1.67E-08 |
Ka-Association rate constant
Kd-Dissociation rate constant
KD-Dissociation binding constant
Table S1. Surface plasmon resonance binding affinity of selected Nanobodies (Nbs) against FMD 3ABC recombinant protein

## Slide 2
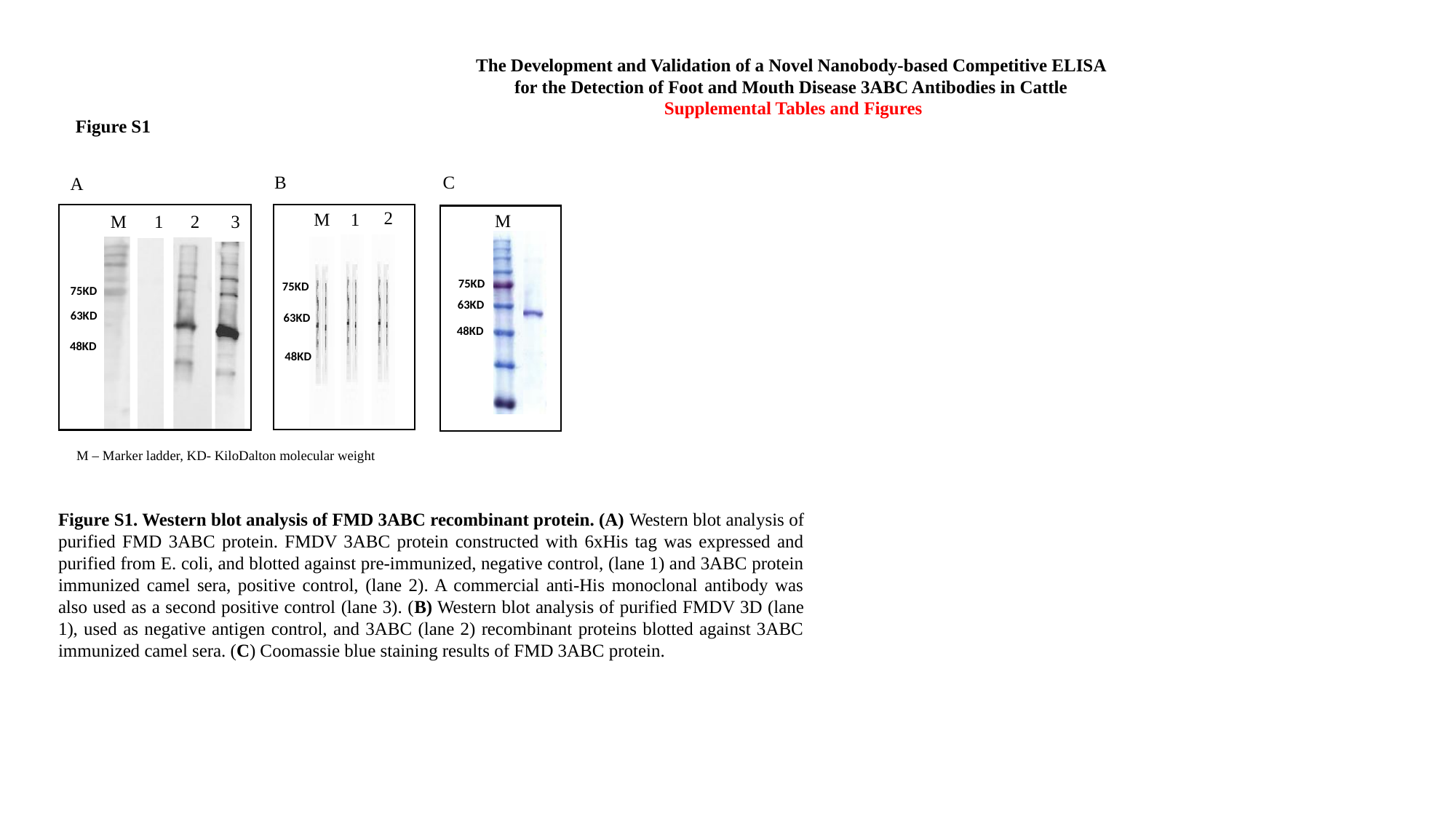

The Development and Validation of a Novel Nanobody-based Competitive ELISA
for the Detection of Foot and Mouth Disease 3ABC Antibodies in Cattle
Supplemental Tables and Figures
Figure S1
C
B
A
2
1
M
M
2
3
1
M
75KD
75KD
75KD
63KD
63KD
63KD
48KD
48KD
48KD
M – Marker ladder, KD- KiloDalton molecular weight
Figure S1. Western blot analysis of FMD 3ABC recombinant protein. (A) Western blot analysis of purified FMD 3ABC protein. FMDV 3ABC protein constructed with 6xHis tag was expressed and purified from E. coli, and blotted against pre-immunized, negative control, (lane 1) and 3ABC protein immunized camel sera, positive control, (lane 2). A commercial anti-His monoclonal antibody was also used as a second positive control (lane 3). (B) Western blot analysis of purified FMDV 3D (lane 1), used as negative antigen control, and 3ABC (lane 2) recombinant proteins blotted against 3ABC immunized camel sera. (C) Coomassie blue staining results of FMD 3ABC protein.

## Slide 3
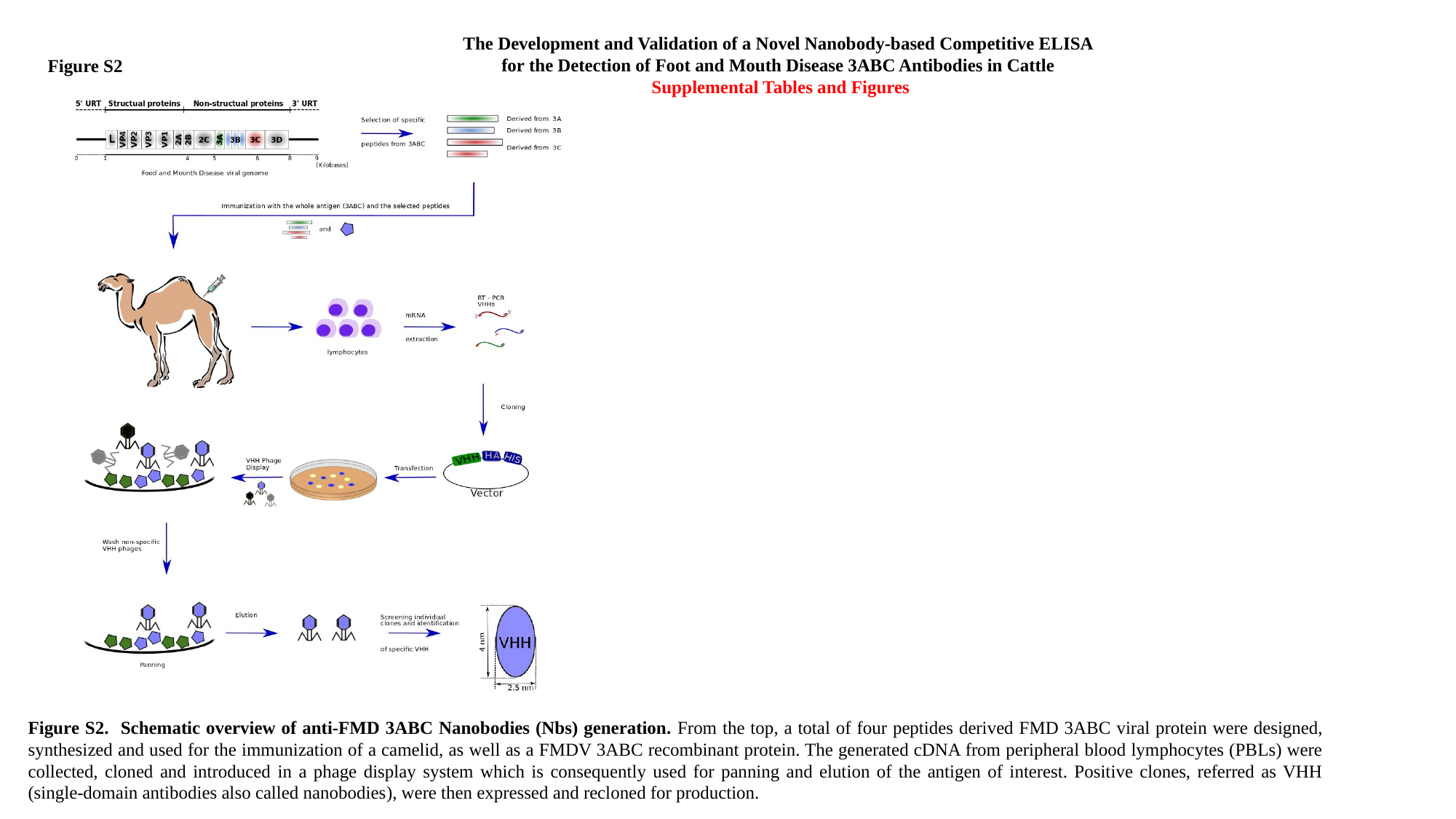

The Development and Validation of a Novel Nanobody-based Competitive ELISA
for the Detection of Foot and Mouth Disease 3ABC Antibodies in Cattle
Supplemental Tables and Figures
Figure S2
Figure S2. Schematic overview of anti-FMD 3ABC Nanobodies (Nbs) generation. From the top, a total of four peptides derived FMD 3ABC viral protein were designed, synthesized and used for the immunization of a camelid, as well as a FMDV 3ABC recombinant protein. The generated cDNA from peripheral blood lymphocytes (PBLs) were collected, cloned and introduced in a phage display system which is consequently used for panning and elution of the antigen of interest. Positive clones, referred as VHH (single-domain antibodies also called nanobodies), were then expressed and recloned for production.

## Slide 4
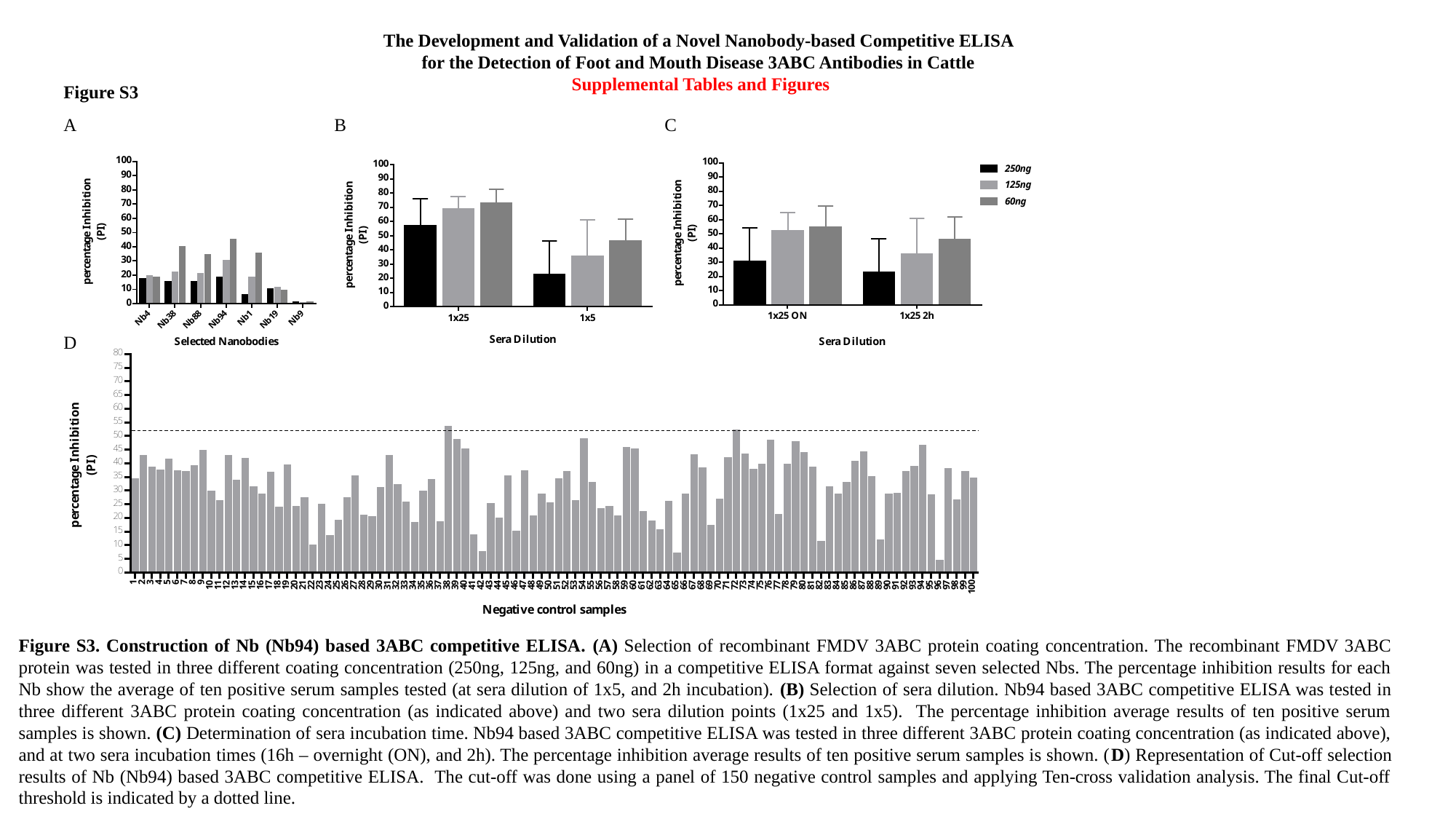

The Development and Validation of a Novel Nanobody-based Competitive ELISA
for the Detection of Foot and Mouth Disease 3ABC Antibodies in Cattle
Supplemental Tables and Figures
Figure S3
A
B
C
D
Figure S3. Construction of Nb (Nb94) based 3ABC competitive ELISA. (A) Selection of recombinant FMDV 3ABC protein coating concentration. The recombinant FMDV 3ABC protein was tested in three different coating concentration (250ng, 125ng, and 60ng) in a competitive ELISA format against seven selected Nbs. The percentage inhibition results for each Nb show the average of ten positive serum samples tested (at sera dilution of 1x5, and 2h incubation). (B) Selection of sera dilution. Nb94 based 3ABC competitive ELISA was tested in three different 3ABC protein coating concentration (as indicated above) and two sera dilution points (1x25 and 1x5). The percentage inhibition average results of ten positive serum samples is shown. (C) Determination of sera incubation time. Nb94 based 3ABC competitive ELISA was tested in three different 3ABC protein coating concentration (as indicated above), and at two sera incubation times (16h – overnight (ON), and 2h). The percentage inhibition average results of ten positive serum samples is shown. (D) Representation of Cut-off selection results of Nb (Nb94) based 3ABC competitive ELISA. The cut-off was done using a panel of 150 negative control samples and applying Ten-cross validation analysis. The final Cut-off threshold is indicated by a dotted line.

## Slide 5
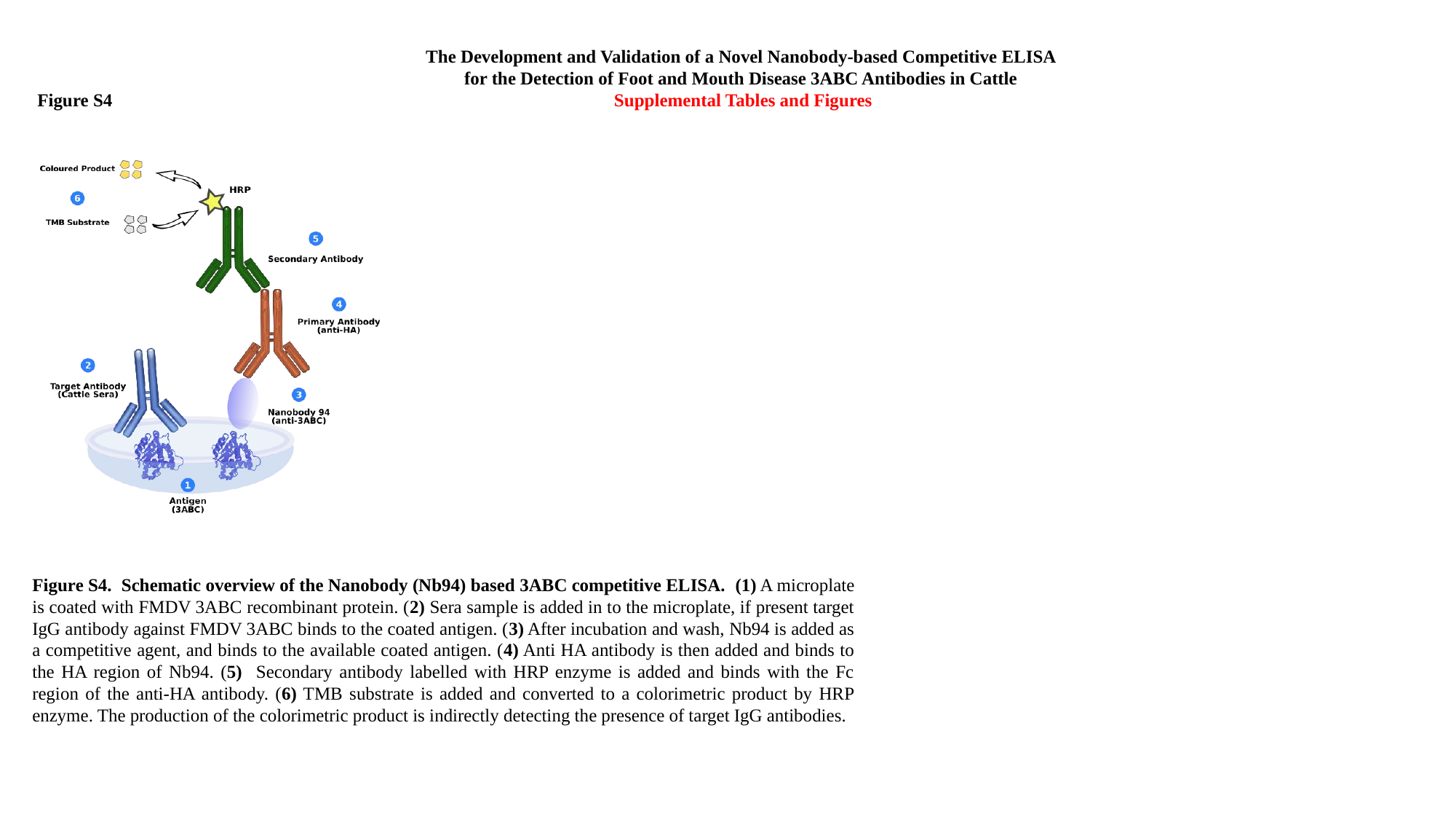

The Development and Validation of a Novel Nanobody-based Competitive ELISA
for the Detection of Foot and Mouth Disease 3ABC Antibodies in Cattle
Supplemental Tables and Figures
Figure S4
Figure S4. Schematic overview of the Nanobody (Nb94) based 3ABC competitive ELISA. (1) A microplate is coated with FMDV 3ABC recombinant protein. (2) Sera sample is added in to the microplate, if present target IgG antibody against FMDV 3ABC binds to the coated antigen. (3) After incubation and wash, Nb94 is added as a competitive agent, and binds to the available coated antigen. (4) Anti HA antibody is then added and binds to the HA region of Nb94. (5) Secondary antibody labelled with HRP enzyme is added and binds with the Fc region of the anti-HA antibody. (6) TMB substrate is added and converted to a colorimetric product by HRP enzyme. The production of the colorimetric product is indirectly detecting the presence of target IgG antibodies.

## Slide 6
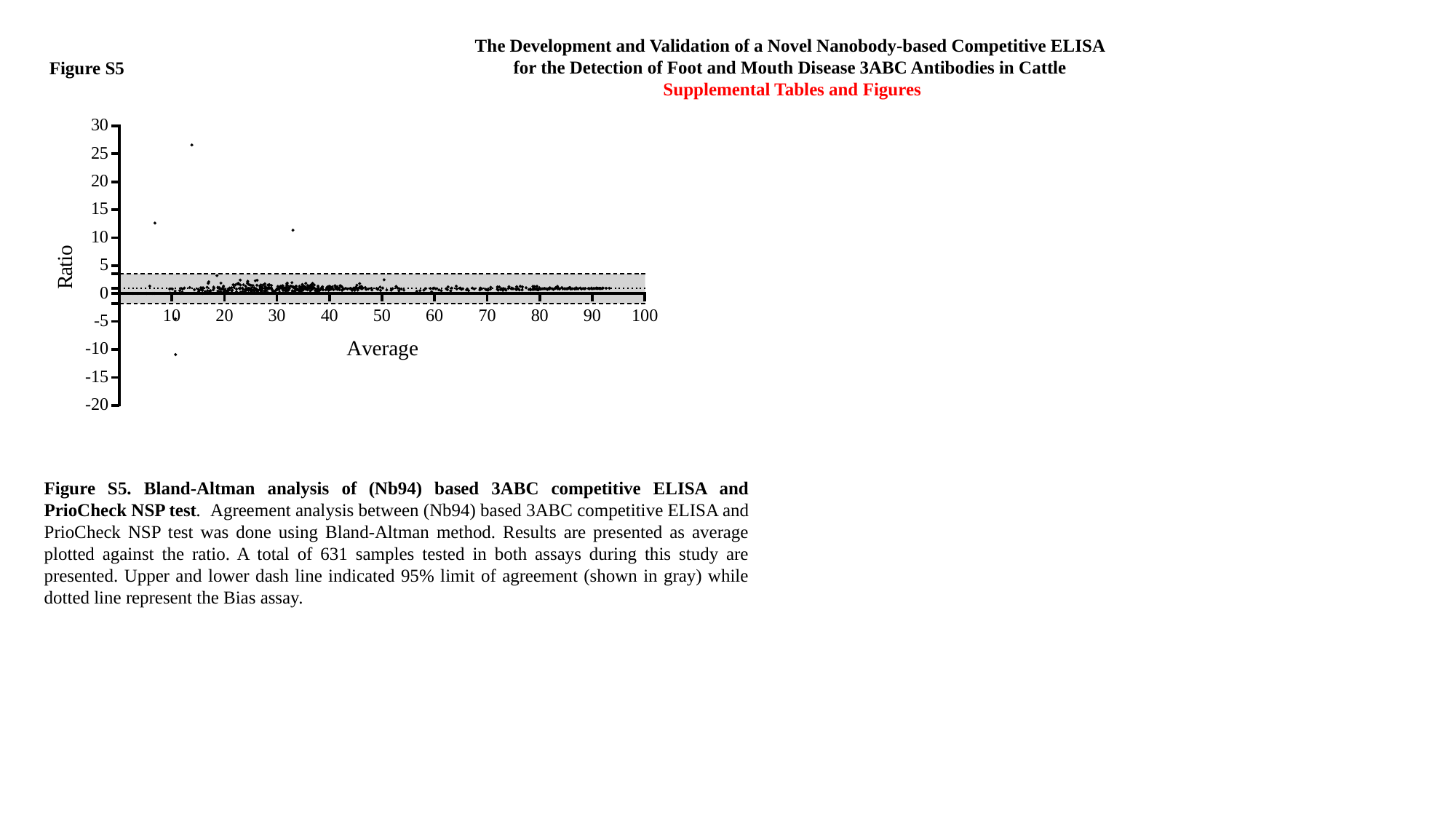

The Development and Validation of a Novel Nanobody-based Competitive ELISA
for the Detection of Foot and Mouth Disease 3ABC Antibodies in Cattle
Supplemental Tables and Figures
Figure S5
Figure S5. Bland-Altman analysis of (Nb94) based 3ABC competitive ELISA and PrioCheck NSP test. Agreement analysis between (Nb94) based 3ABC competitive ELISA and PrioCheck NSP test was done using Bland-Altman method. Results are presented as average plotted against the ratio. A total of 631 samples tested in both assays during this study are presented. Upper and lower dash line indicated 95% limit of agreement (shown in gray) while dotted line represent the Bias assay.
